# Detecting similar binding pockets to enable systems polypharmacology

**DOI:** 10.1371/journal.pcbi.1005522

**Published:** 2017-06-29

**Authors:** Miquel Duran-Frigola, Lydia Siragusa, Eytan Ruppin, Xavier Barril, Gabriele Cruciani, Patrick Aloy

**Affiliations:** 1 Joint IRB-BSC-CRG Program in Computational Biology, Institute for Research in Biomedicine (IRB Barcelona), The Barcelona Institute of Science and Technology, Barcelona, Catalonia, Spain; 2 Molecular Discovery Limited, London, United Kingdom; 3 Department of Computer Science & Center for Bioinformatics and Computational Biology, University of Maryland, College Park, Maryland, United States of America; 4 School of Computer Sciences, Tel Aviv University, Tel Aviv, Israel; 5 Department of Physiology and Pharmacology, Sackler Faculty of Medicine, Tel Aviv University, Tel Aviv, Israel; 6 Departament de Fisicoquímica, Facultat de Farmàcia, Universitat de Barcelona, Barcelona, Catalonia, Spain; 7 Institució Catalana de Recerca i Estudis Avançats (ICREA), Barcelona, Catalonia, Spain; 8 Department of Chemistry, Biology and Biotechnology, University of Perugia, Perugia, Italy; Icahn School of Medicine at Mount Sinai, UNITED STATES

## Abstract

In the era of systems biology, multi-target pharmacological strategies hold promise for tackling disease-related networks. In this regard, drug promiscuity may be leveraged to interfere with multiple receptors: the so-called polypharmacology of drugs can be anticipated by analyzing the similarity of binding sites across the proteome. Here, we perform a pairwise comparison of 90,000 putative binding pockets detected in 3,700 proteins, and find that 23,000 pairs of proteins have at least one similar cavity that could, in principle, accommodate similar ligands. By inspecting these pairs, we demonstrate how the detection of similar binding sites expands the space of opportunities for the rational design of drug polypharmacology. Finally, we illustrate how to leverage these opportunities in protein-protein interaction networks related to several therapeutic classes and tumor types, and in a genome-scale metabolic model of leukemia.

## Author summary

Traditionally, the fact that most drugs are promiscuous binders has been a major concern in pharmacology, due to the occurrence of undesired off-target clinical events. In the recent years, however, the realization that many diseases are the result of complex biological processes has encouraged rethinking of drug promiscuity as a promising feature, since it is sometimes necessary to interfere with multiple receptors in order to overcome the robustness of disease-related networks. One way to identify groups of proteins that could be targeted simultaneously is to look for similar binding sites. We have massively done so for all human proteins with a known high-resolution three-dimensional structure, unveiling a vast space of ‘polypharmacology’ opportunities. Of these, we know, a great majority is not of therapeutic interest. To pinpoint promising multi-target combinations, we advocate for the use of computational tools that are able to rapidly simulate the effect of drug-target interactions on biological networks.

## Introduction

Multi-target strategies are a natural approach to tackling complex diseases. A fine way to achieve a multi-target effect is through drug polypharmacology, i.e. the simultaneous modulation of several targets by means of one single agent [1, 2], which poses pharmacokinetic advantages over drug combinations [3]. In the light of systems biology, it seems reasonable to first select a combination of receptors that will modify the biological network as desired, and then design a ligand that it is able to simultaneously bind them [3]. Unfortunately, in practice, most target combinations that are identified in the network analysis step will not show cross-pharmacology, since the discovery of intended promiscuous drugs is still restricted to members of the same protein family [4]. Besides few remarkable exceptions [5–9], the rational molecular design of ligands that intentionally bind several unrelated proteins is far too complicated, yielding ambivalent, non-drug like molecules.

Although challenging to achieve rationally, polypharmacology is a recognized feature of many approved drugs [10], and even those molecules praised to be highly specific, like imatinib, end up eliciting a quite rich interaction profile [11]. This unavoidable promiscuity has long been regarded as detrimental due to adverse off-target reactions [12, 13], but at the same time it paves the way to a reverse drug design strategy, where one would first massively look for proteins that are likely to bind the same ligand, and only then do network analysis to identify the small fraction of putative target combinations that are of therapeutic interest.

A systematic way to detect pairs of proteins that could share a ligand is to compare binding sites in their 3D structures [14, 15]. Arguably, the design of ligands that dock to similar pockets is simpler and more suited to the current medicinal chemistry toolbox, and binding site characterization and comparison methods have flourished with this aim [16]. Using these methods, today it is possible to identify alternative drug targets [17], predict molecular functions [18] and uncover links between remote proteins [6]. Surprisingly, though, there is a lack of bona fide systems pharmacology continuations of the binding site comparison approach, and it remains unclear whether the space of cross-pharmacology uncovered by structural analysis will ultimately be useful to yield relevant impact on large biological networks.

To address this question, we have applied binding site similarity analysis in several systems biology scenarios. For this, we have exhaustively compared pockets across a large fraction of the human proteome, finding connections between close and distant proteins belonging to families with varied tradition in drug discovery. Then, inside the rich collection of polypharmacology opportunities, by applying systems biology techniques, we have pinpointed those cases that could have an impact on protein-protein interaction networks (PPIs) related to several therapeutic areas and tumor-types [19], and to a genome-scale metabolic model (GSMM) of cancer cell lines [20].

## Results and discussion

### The space of structural cross-pharmacology is vast

**Fig 1:**
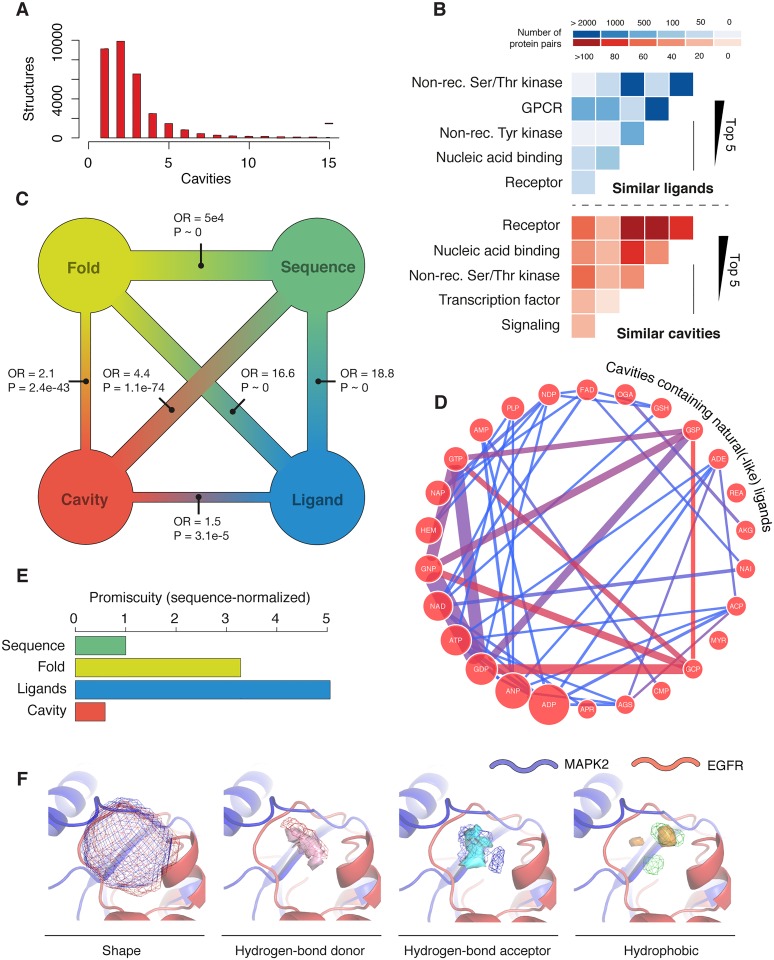
The space of cross-pharmacology. (A) Number of cavities per structure (only structures with at least one cavity are included). (B) Top-5 families emerging from a ligand- (blue) and structure-centered (red) protein comparison. Darkness of the cells quantifies the number of protein pairs. (C) Protein-protein similarity based on cavities, fold, sequence and ligands. OR refers to the enrichment odds ratio of the comparison between two similarity spaces, accompanied with its P-value. Larger ORs correspond to more coincidence of pairs; for instance, as expected, most pairs that have a similar sequence also have a similar fold. (D) Cross-pharmacology of cavities containing ubiquitous natural(-like) ligands in the PDB. In the diagram, an edge is drawn between two ligands if a pair of similar cavities containing each of them is found, being the width of the edge proportional to the pairs of proteins where this happens. Notice, e.g., the cluster formed by GTP, GDP, GSP and GCP, or by ATP-like or NAD-like molecules. Size of the nodes is proportional to the number of unique structures where the ligand is bound in a cavity. Only ligands found in at least 5 proteins are shown. (E) Specificity of protein cross-pharmacology, relative to the promiscuity based on sequence similarity. Please note that these plots are sensitive to the choice of similarity cutoffs. Rather than represent optimality to capture polypharmacology, these cutoffs were chosen to exemplify commonly taken thresholds in the scientific literature. (F) Allosteric MAPK2 and EGFR cavities, superposed. Shape, hydrogen bond donor, acceptor and hydrophobic patterns are shown.

In order to navigate the space of putative binding sites in human proteins, a fast and automated protocol is necessary. We used BioGPS [14], which first characterizes cavities using molecular interaction fields, and then summarizes them with quadruplet fingerprints. We found 87,300 cavities (Fig 1A) in 31,900 protein chains from 3,700 unique proteins. Then, we performed a pairwise pocket comparison. Pairs of cavities with a BioGPS score above 0.6 were classified as ‘similar’. This threshold was tuned while we were developing BioGPS, five years ago. Thereafter, we’ve tested it in other datasets, proving its validity to capture polypharmacology [15, 21]. Reassuringly, when analyzing co-crystallized ligands in the PDB, we observed that pairs of pockets above this cutoff indeed tend to accommodate the same ligands (S1A–S1C Fig), being cavities able to embed the totality of the ligand (S1D Fig) while remaining relatively small (S1E Fig) and highly specific for ligand-binding regions (>40% of the cavities overlap with ligands). In addition, our cavity pairs are able to account for experimental cross-pharmacology, such as that observed in kinase inhibition screens (S1H Fig) [22], or in closely related natural products (Figs 1D and S1I). Similarly, we found that targets of the same drug tend to display similar pockets (odds ratio OR = 4.02, P = 2.9·10^−48^), proving the pharmacological relevance of the pockets identified and compared by BioGPS.

In total, we discovered 181,500 pairs of similar cavities, 68.8% of them corresponding to pockets in different structural instances of the same protein, and the other 31.2% to cavities in distinct proteins. Corresponding cavities in different structures of the same protein have remarkably high BioGPS scores, proving the sensitivity of this similarity measure (S1G Fig). The fact that most of the paired cavities may be matched to another structural instance of the same protein or fold (S1F Fig) demonstrates that, while sensitive, the similarity measure is robust to small structural fluctuations and variations. Overall, 23,148 pairs of proteins shared at least one putative binding site (see Supporting S1 Data). From this structural standpoint, the average protein was related to about 7 other proteins, some of which are structurally and functionally unrelated (Fig 1B and 1C), illustrating the known degeneracy of protein binding sites [23]. To demonstrate the power of BioGPS, in Fig 1F we focus on two *allosteric* cavities that we found in the Epidermal Growth Factor Receptor (EGFR) and the Mitogen-Activated Protein Kinase 2 (MAPK2). While distant in the kinase phylogeny, off-target interactions between EGFR and MAPK2 have been previously detected in high-throughput kinase inhibition screens [24, 25]. The allosteric pockets that we found, when superimposed, show similar shape and non-bonded hydrophobic and polar patterns (S1F Fig), hinting towards a structural hypothesis for the MAPK2-EGFR cross-pharmacology.

### Similar cavities can be found among proteins with no apparent relationship

Due to their functional relevance, binding sites are under higher evolutionary pressure than the rest of the protein structure [26]. Thus, similar cavities can be found between apparently unrelated proteins. However, even if strongly conserved, the sites need to confer specificity within the same family, as it is well known for kinases [27]. These two aspects are apparent from our results. While, in general, sequence-related proteins tend to share cavities (P = 2.1·10^−74^), we could find many examples of far-off proteins whose pockets are alike, and of close sequences with divergence in the binding site (see the modest odds ratios in Fig 1B). The same trend could be observed at the fold level (P = 2.4·10^−43^), detecting analogous pockets in distinct folds, while sometimes failing to match pockets inside the same structural family. Together, these results outline an optimal scenario for the multi-targeting idea, suggesting that more than one function can be perturbed at a time, and also that specificity can be achieved among functionally related proteins.

### Polypharmacology opportunities remain largely unexplored

Over the years, the functional connection of proteins has introduced biases in the chemical libraries screened in binding assays [28]. Drug discovery is eminently incremental, and chemotypes known to bind one receptor are often tested in closely related proteins [29]. Chemogenomics databases now collect ligands for about three thousand human proteins [30], and these ligands can be used to describe targets from a chemical viewpoint [31]. Accordingly, two proteins are associated if they globally recognize similar ligands, providing valuable guidance for the eventual molecular design of polypharmacology. This approach has been successful so far [32], but it inherits some major shortcomings, namely the strong dependence on the amount and quality of pharmacological data [33], and the aforementioned bias introduced by incremental library design.

In order to evaluate the overlap between the ligand- and structure-centered views of cross-pharmacology, we have used an in-house version of the similarity ensemble approach (SEA) developed by Keiser et al. [34], a popular method to relate proteins through the chemistry of their ligands (S2 Fig). In general, and as a further proof of the relevance of cavity comparisons, we observed a significant correspondence between ligand- and cavity-based similarities (P = 3.1·10^−5^). Even if so, the two cross-pharmacology spaces differed in many aspects. Given the biases in chemogenomics data, the chemocentric view correlates more strongly with sequence and fold relatedness (P ~ 0, Fig 1C). As it can be seen in Fig 1B, proteins in the same family shared similar ligands, being kinases and G-protein coupled receptors (GPCRs) the most prominent examples due to their pharmaceutical importance. On the contrary, the structural viewpoint was poorly applicable to GPCRs, for which 3D-crystal data are scarce [35], and highlighted other receptors and nucleic acid binding proteins instead (Fig 1B). Overall, it seems that cavity comparisons offer a complementary catalogue of protein pairs with new, less trivial inter-family opportunities at the expense of certainty in the pharmacology, since ligand binding assay data are not always available for the candidate receptors.

While the space of cross-pharmacology opens wide when we focus on binding sites, we observe a drastic decrease in its density: in relative terms, given a protein we found less partners by inspecting cavities than sequences or folds, and far less than from the ligand viewpoint (Fig 1E). This demonstrates the specificity that can be achieved at the binding pocket level, and suggests that structure-based polypharmacology could enable a fine-tuned promiscuity, i.e. a good selectivity in the eventual systems-level chemical-protein interactome.

### Structure-based simultaneous putative targets reach distinct regions of the human interactome

To achieve systems-level interactions, multi-target agents should be able to modulate more than one molecular function. As suggested above, structural cross-pharmacology facilitates this feature, which has been classically achieved through drug combinations [36–38]. Despite some major difficulties, the asset of drug combinations is that, in principle, no restrictions exist to modulate multiple cellular processes.

**Fig 2:**
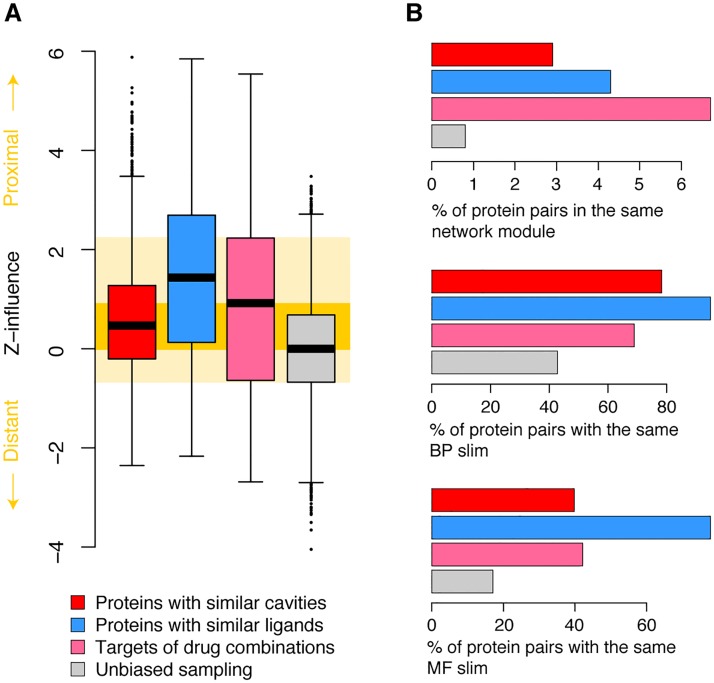
Distribution of multi-target opportunities in the binary interactome. (A) Network-based influence between proteins with similar cavities (red) and ligands (blue), compared to the background influence of random proteins in the interactome (gray). The relative influence between targets in drug combinations is plotted in purple. The distributions include groups with up to five comparisons, and the maximum influence among all of the pairs in the group was taken as the representative; in other words, closest pairs were picked in those drug combinations that involved more than one target per drug, and in cavity and ligand comparisons that brought together more than two proteins. To correct for the group size, we defined a Z-score by sampling 10,000 random groups in the size range. The orange shape spans the area between the quartiles of drug combinations and the unbiased sampling of nodes. (B) Characteristics of protein pairs that could be targeted simultaneously (polypharmacology opportunities) or that are used in successful drug combinations. In the upper plot, proportion of pairs of protein belonging to the same topological module in the network (as defined by the overlapping cluster generator [42]); in the middle plot, pairs of proteins with the same biological process (BP) broad (‘slim’) term [70]; similarly, in the bottom plot, pairs of proteins with the same molecular function (MF). For simplicity, in the case of drug combinations, only those with one target per drug are included here.

While largely incomplete, the human binary interactome sketches a map of these cellular processes, and can be used to unravel their crosstalk [39]. In the context of the human interactome, targets of successful drug combinations are close to each other (Fig 2A). This relative proximity is in part due to the pathway-based mindset applied to drug combination discovery [40], and also reflects that, in order to be effective, a multi-target intervention should interplay within the network. Because of its inherent functional bias, ligand-based similarity offers combinations of targets that are even more local than the standards of efficacious drug combinations [41] (Fig 2A). On the contrary, structure-based cross-pharmacology opportunities, while still closer in the network than the background expectation resulting from an unbiased sampling, are further apart. Likewise, beyond measuring pairwise network influence, modules can be encircled in the interactome to reveal communities of proteins devoted to a certain biological process [42]. We have seen that, in general, targets of drug combinations act in few modules, and that groups of proteins with structural cross-pharmacology are more disperse (Fig 2B). In turn, the functional diversity achieved by drug combinations and proteins with similar cavities is comparable, and greater than the diversity obtained from the chemo-centric viewpoint (Fig 2B). It appears, thus, that polypharmacology opportunities based on binding site similarity are widespread enough to provide functional variety, but it is clear that, at least from the network viewpoint, many multi-target opportunities do not resemble the successful ones achieved through drug combinations. The space of structural cross-pharmacology is vast: to detect the small portion of polypharmacology opportunities that will be of therapeutic interest, automated systems biology setups are necessary [43].

### Polypharmacology to enhance existing therapies

**Fig 3:**
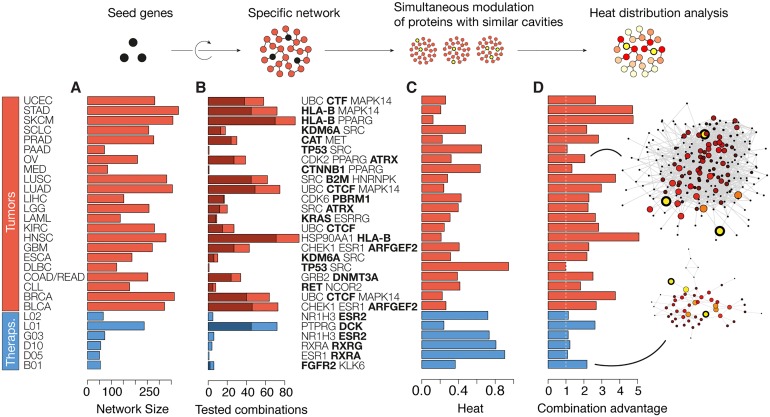
Global influence of structural polypharmacology on therapy- and tumor-specific networks. (A) Size of the networks. (B) Number of multi-target simulations performed in each network; the shade depicts the proportion of these that have an advantage >25% over the best individual seed interference. (C) Heat distribution, relative to the mild, ideal distribution of heat across nodes. (D) Advantage of the combination, compared to the best single seed (gene in bold in the list). The B01 and OV networks are laid out. Perturbed nodes are highlighted with a thick line, and heat is proportional to the size of the nodes (log scale). See [19] for tumor-type abbreviations. The ATC codes displayed refer to endocrine therapies (L02), antineoplastic agents (L01), sex hormones and modulators of the genital system (G03), anti-acne preparations (D10), antipsoriatics (D05) and antithrombotic agents (B01).

In the dawn of systems pharmacology, several approaches have been proposed to identify those groups of targets that will cooperate to elicit the desired therapeutic effect [44, 45]. Following the module-specificity observed for drug combinations, one possibility is to focus on those regions of the interactome that are related to a phenotype of interest, and then do network analysis on these specific regions to prioritize multi-target opportunities. To exemplify this approach, we have collected drug targets in several therapeutic categories and built sub-networks for each of them [46]. These therapy-specific networks will only be sound if the corresponding drug targets are strongly connected, i.e. if the interactome is capturing the underlying biology of the therapy. This was the case for six major therapeutic classes (P < 0.01, Fig 3). The resulting networks grown around approved drug targets included a median of 60.5 proteins (Fig 3A), and were thus appropriate for sensitive network modulation analysis [47, 48] (see Methods and S3 and S4 Figs for further details on the construction of these networks).

Since therapy-specific networks are seeded with known therapeutic effectors, a reasonable assumption is that node ablations are bound to a beneficial response. With this premise, a way to prioritize protein combinations is to measure their global impact on the network, i.e. to evaluate the effect of each node on the rest of the proteins. In the context of disease mutations, this feature has been successfully modeled by heat flow analysis [49]. Analogously, in each network, we simultaneously perturbed (assigned heat to) protein sets with structural cross-pharmacology (i.e. sets of proteins that all of them have cavities similar to one or more of the proteins in the group, and where at least one cavity is similar to cavities in all of the other proteins) (Fig 3A). Those target combinations that best spread the heat were prioritized. While many combinations had no major advantage over single targets, in two of the six networks it was possible to find a multi-target case with a marked systems level clout (Fig 3D). In the therapeutic network related to antithrombotic agents (ATC: B01), for instance, the simultaneous inhibition of the known target FGFR2 and KLK6 was predicted to exert an effect on the network twice as great as the best individual target, requiring half of the heat, i.e. less inhibition strength, in each of the nodes.

### Multi-target modulations have broad impact on tumor-specific interactomes

While intimately related to a therapeutic response, the therapy-specific networks above cannot go beyond current medicinal knowledge, since they are based on the available drug repertoire. More commonly, systems pharmacology departs from a disease-specific network [50], and target combinations are mined therein. Yet, in the context of PPIs the connection between changes in a disease-specific network and the eventual therapeutic effect is poorly understood. Perhaps an exception is cancer, where the desired outcome is cell death, which can be roughly modeled as a global impact on the network; arguably, this impact is captured by the heat distribution method applied above [49]. To confirm the therapeutic relevance of the measure, we computed the heat released by known targeted therapies on the corresponding tumor types, and observed a significantly high heat circulation (S3D Fig, P < 10^−4^).

Of the 34 tumor types considered [19], 22 had significantly connected driver genes (P < 0.01), and we focused on these tumors to build the tumor-specific networks (median size of 264 proteins). Following the assumption that good candidate therapies will prominently distribute heat in the tumor network, we screened our polypharmacology opportunities and observed that, for most (20) tumor types it was possible to detect at least one combination that was able to distribute heat at least 25% better than any of the corresponding individual seed (on average, 2.7-fold advantages could be achieved) (Fig 3D). In some tumor types, like stomach adenocarcinoma (STAD), skin cutaneous melanoma (SKCM) and heat and neck squamous cell carcinoma (HNSC), the advantage of the combination reached the 5-fold. In Fig 3 we display the network of ovarian cancer, which is representative in terms of size and sensitivity to multiple targets. Here, the simultaneous interference with CDK2, PPARG and ATRX yields a global heat distribution of the 32%, and a 2.1-fold advantage over the ATRX driver gene modulation.

### A metabolism-related combination to selectively reduce proliferation of cancer cell lines

As exemplified above, PPI networks are widely applicable, yet mostly descriptive. A less generic, but more predictive, systems biology tool is genome-scale metabolic modeling (GSMM) [51]. These models unprecedentedly link the properties of the network to phenotypic traits [52], yielding very informative simulations compared to the tentative measures of network impact that can be obtained from protein interactomes. Interference with single metabolic genes or reactions are routinely quantified in GSMMs, and have been shown to mimic drug inhibition [20]. However, the brute-force simulation of multi-target effects is impractical, especially beyond double inhibitions, due to combinatorial explosion. In this sense, the constrained space of cross-pharmacology drastically reduces the number of combined inhibitions to simulate, offering a viable corpus with enriched properties for drug design.

We illustrate this advantage by considering a GSMM of leukemia cell lines and one of healthy leukocytes [20]. Both models are based on the same reconstruction of the human metabolism and only differ in the flux bounds of the reactions. We could collect structures for 567 of the 1,878 (30.2%) metabolic proteins, and find cavities in a large proportion of them (70.2%), as expected given their interplay with endogenous small molecules. Moreover, we detected a marked cross-pharmacology between proximal metabolic proteins (OR = 4.43, P < 10^−4^), where the product metabolite of the first yields the substrate of the second.

To identify promising polypharmacology, we simulated the concurrent inhibition of proteins with similar cavities, and measured the impact on biomass production and metabolic cancer hallmarks, like high lactate secretion or anoxia [53]. Biomass production correlates well with proliferation rates [20]. Thus, a multiple inhibition causing larger reduction of biomass in the cancer GSMM than in the healthy one is both effective and selective. If, in addition, the decrease in biomass production cannot be achieved by silencing the individual genes, then the multiple inhibition corresponds to genuine polypharmacology.

**Fig 4:**
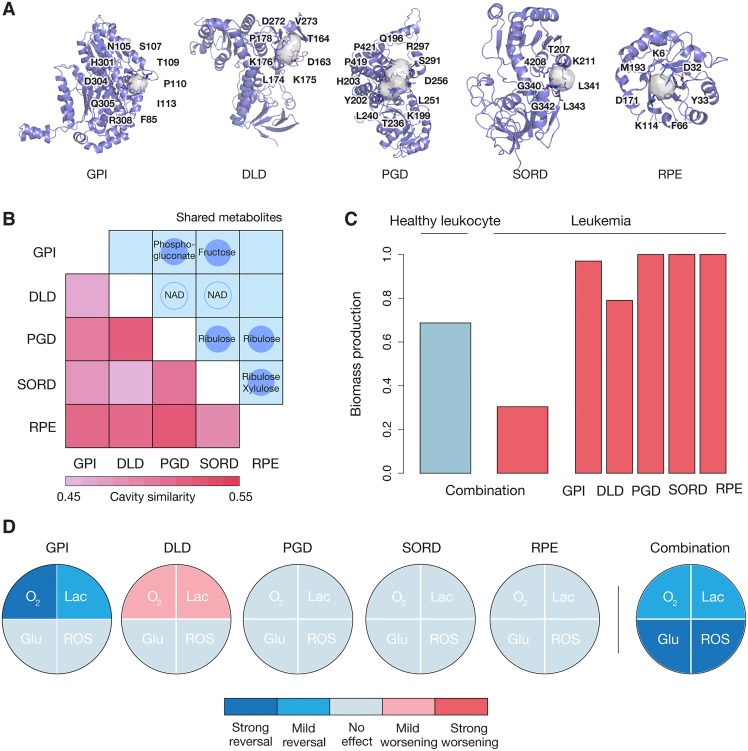
A selective target combination. (A) Structures of GPI (PDB ID: 1iri, chain C), DLD (PDB ID: 1zmc, chain C), PGD (PDB ID: 2jkv, chain D), SORD (PDB ID: 1pl8, chain C) and RPE (PDB ID: 3ovp, chain C) are displayed, together with cavity residues. Please note that these cavities are representative, as several structures exist for each of the proteins. For a deeper exploration, please refer to Supporting S1 Data. (B) Best similarity between cavities in GPI, DLD, PGD, SORD and RPE. Highest similarities represent, in principle, easier cases of polypharmacology design. In the upper triangle, the main metabolic chemotypes related to the enzymes are also displayed. (C) Reduction of biomass production upon the simultaneous inhibition in cancer (red) and normal (blue) cell lines. The effect of individual inhibitions in also showed. (D) Influence of each inhibition on metabolic cancer hallmarks. O_2_ stands for oxygen consumption, Lac for lactate secretion, Glu for glucose uptake, and ROS for reactive oxygen species production. The assignment of ‘strong' and ‘mild’ reversals was based on visual inspection of the maximal flux of the corresponding reactions. When the maximal flux approached (>50% of the difference) the healthy cell line, it was classified as ‘strong reversal' ('strong worsening' if it otherwise diverged); `mild' effects were assigned to effects of less than 50%; ‘no effect' was assigned when one could observe essentially no change.

Due to the limited structural coverage of the metabolic network, and to obtain a sufficiently long list of protein combinations to screen, we applied milder constraints on the inter-similarity of cavities (see Methods). In Fig 4A, we present five proteins (GPI, DLD, PGD, SORD, and RPE) whose simultaneous inhibition could produce a selective decrease of proliferation rates in leukemia cell lines Fig 4C. Additionally, we observed in the simulation that metabolic cancer hallmarks were reversed upon the multiple inhibition. Glucose uptake, lactate secretion, and production of reactive oxygen species (ROS) were reduced, while oxygen consumption was increased (Fig 4D). None of the single inhibitions was able to produce such a widespread effect.

It is out of the scope of this work to further explore the feasibility of simultaneously targeting these five proteins, which are mostly involved in the metabolism of carbohydrates and share chemotypes in their metabolites, like the fructose and ribulose scaffolds (Fig 4B) [54]. In order to discover more combinations, ideally with yet stronger cavity similarity, and to ensure the inhibitory effect of the binding event, deeper structural annotation of metabolic reconstructions will be necessary. This claim has also been raised for other types of biological networks [12], highlighting the importance of keeping structural details in the systems era.

### Concluding remarks

The emergence of systems biology in the last decade has brought about new therapeutic opportunities. Among them, the idea of exploiting drug promiscuity to exert systems-level effects is particularly compelling, but its feasibility remains unclear. The few examples of intended polypharmacology are usually within protein families [4], and, traditionally, the discovery of alternative targets has been applied to drug repositioning instead of holistic therapies [55]. The reason for this is that most target combinations are not of therapeutic value, making it very unlikely to hit interesting target profiles if only a few closely related proteins are inspected.

To overcome this issue, structural biology offers a systematic means to explore the space of protein cross-pharmacology by detecting and comparing putative binding sites [16]. We have seen that proteins with similar binding pockets are scattered all over biological networks, holding promise for enabling systems pharmacology. Our analysis has uncovered a large amount of multi-target opportunities to be screened against cellular networks, and the exploratory simulations in different systems biology scenarios are very encouraging. However, there is a long agenda for drug polypharmacology. We lack full structural coverage in most biological systems [56, 57], sometimes missing relevant drug targets, or having only partial structures (S5 Fig). Even if this gap can be diminished with homology models [58], it is not clear whether the corresponding binding sites will be accurate enough, making it critical to develop integrative methods that are able to account for homologs from other species. Further, although structural cross-pharmacology offers *a priori* a good drug design scenario, it is not yet clear if the rational design of polypharmaceuticals is feasible at large: detecting similarity of cavities is only the first step of an artisanal discovery process to identify molecules that are suitable as drugs [59, 60], keep the right balance of potency across targets [61] and do not compromise their off-target selectivity. In this work, we have focused on protein structures, and only slightly explored the ligand side. In the light of our results, we envision that methods that combine structural and ligand information will help alleviate the intrinsic limitations of the structural viewpoint [62, 63].

Equally critical is the dearth of methods to prioritize target combinations among the enormous pool of possibilities. Thus far, no blueprint exists to use protein interaction networks for predictive means, and more quantitative systems biology frameworks, like metabolic models, are only applicable to certain diseases. Nowadays, active research is addressing these problems. In our opinion, the discovery of multi-target therapies can be empowered by the constrained sampling of combined effectors, and it is our belief that efforts put on fast and automated protocols will be key to finally shift pharmacology to the systems level.

## Materials and methods

### Cavity search, description and comparison

We collected experimental protein structures from the human interactome available in Interactome3D [58], considering each chain separately. Then, we used BioGPS to perform the structural analysis of putative binding pockets. BioGPS has three steps: (1) cavity search using FLAPsite, (2) cavity description using GRID [64] molecular interaction field potentials, and (3) cavity comparison [14]. We performed an all-vs-all comparison of all detected cavities, and considered those cavities with a BioGPS score above 0.6 as ‘similar’. This score is recommended by the BioGPS developers, and we have confirmed its validity by analyzing the enrichment of cavity pairs containing the same ligand (S1A and S1B Fig), and the best compromise between precision and recall (S1C Fig).

### Ligand-based similarity of proteins

To calculate the ligand-based similarity of proteins, we did an in-house implementation of SEA using molecules in ChEMBL (v19) with affinity below 10 uM [34]. After analyzing background data, we found that a Tanimoto cutoff of 0.55 optimally fitted an extreme-value distribution instead of a Gaussian curve (FP2 fingerprints). S2A–S2C Fig shows the background adjustments; E-values of ligand set similarities were calculated therefrom. We chose 10^−4^ as an E-value cutoff. This cutoff was used in a study to detect off-targets [32], and we have seen that, indeed, it captures pairs of proteins containing the same ligand (OR = 18.7, P ~ 0). In order to confirm that SEA is a good representative of ligand-based similarity, we performed in parallel a Naïve Bayes (NB) multi-target virtual screening, this time with Morgan fingerprints. This approach is, in nature, very different to SEA. Reassuringly, NB and SEA trends were strongly correlated, as it can be seen in S2D–S2F Fig.

### Sequence and fold similarity of proteins

To calculate sequence similarity of proteins, we applied JackHMMER with default parameters [65], E-value < 10^−4^. As for the fold annotation of structures, we used the classification of ECOD (January 2015) at level 4 of the hierarchy [66].

### Drug combinations and global network analysis

For the analysis of drug combinations, we considered those classified as ‘approved' or ‘clinical' in DCDB (v2) [41], excluding ‘non-efficacious’ cases. Targets from DCDB were also extracted and used for the interactome-based analysis of the combinations. Influence between pairs of proteins was determined using a pre-computed influence matrix, obtained with a PageRank-like algorithm with a default β of 0.5. When more than one protein was to be compared, we parsimoniously took the pair with the highest influence as representative. Normalization for group size was achieved with 10,000 random groups at each size, and the mean and standard deviation of these background sets were calculated to derive a Z-influence score so that the background had a mean of 0 and a standard deviation of 1.

To analyze topological clusters, the human interactome was submitted to the overlapping cluster generator (OCG) [42]. Slim BP and MF annotations were obtained from GO (January 2015).

### Therapy- and tumor-specific interactomes

In the therapy-specific networks, seeds were obtained from DrugBank (v3) targets [67]; and in the tumor-specific networks drivers were fetched from IntoGen [19]. To select only those cases with a significant interconnectivity, we used the method based on percolation theory described by Menche et al. [68], applying a significance cutoff of P < 0.01 on the size of the largest connected components.

For cases with significant seed connectivity, we built specific networks by sequentially including non-seed (candidate) proteins and extracting the corresponding subgraphs from the interactome. Nodes were included following the DIAMOnD score [46], which is again based on percolation theory. To automatically determine the number of nodes to include, we performed a 10-fold cross-validation on the seeds, i.e. we removed seeds from the seed set and checked (1) whether they were up-ranked (recalled) by DIAMOnD, and (2) whether their inclusion was helping in building a big connected component that gathered a large number of seeds (Fig 3C and 3D). We cut at the mean point of flattening of the two recall curves.

### Measurements of network heat

We used Hotnet (v2) to measure heat propagation on the networks [49]. In a first step, Hotnet calculates an influence matrix from the network. We did so for each of the therapy- and tumor-specific networks. For each network, we optimized the β parameter of Hotnet as recommended by the authors, i.e. by maximizing the average influence at the inflection point of the cumulative distribution of level-one neighbors of nodes of random and topologically varied nodes. In most networks, β ranged from 0.4 to 0.6.

In each modulation, 1,000 heat units (h.u) were arbitrarily assigned. Therefore, in a single modulation, the target protein contained all of the initial heat, e.g. while in a three-node interference, 333 h.u. were given to each node. Qualitatively, this means that in a three-node intervention simulated inhibitions are considerably milder. The area under the cumulative heat distribution, after running Hotnet, was used to measure the global heat. This area was normalized by the ideal heat distribution, obtained by gently (1,000/n h.u.) heating the n nodes of the network. The advantage of a combination over single interference was measured by dividing the global heat of the combination by the best global heat of the individual components. Details are given in S4 Fig.

### Genome-scale metabolic models of cancer

To model the effect of multiple inhibitions on leukemia metabolism, we used the PRIME metabolic models, which are based on the NCI-60 (cancer) and HapMap (healthy) cell line panels [20]. In the PRIME collection, we arbitrarily took the first leukemia cell line of the NCI-60 and the first cell line of the HapMap panels. We modeled inhibitions at the gene level by reducing to the 1% the V_max_ of the corresponding reactions, with the OptKnock method[69]. Reaction rules (AND and OR) of the PRIME models were appropriately taken into account to translate gene inhibitions to the reaction level.

In the OptKnock flux variability analysis, we focused on the biomass reaction, and also on other reactions that are representatives for metabolic cancer hallmarks, e.g. oxygen consumption or reactive oxygen species production. In the flux variability analysis of these reactions, we put as a constraint the maintenance of 80% of the wild-type biomass production.

## Supporting information

S1 Data Pairs of proteins with similar cavities, together with cavity specifications, are available at the file SuppData1.xlsx. (XLSX)

S1 Fig Analysis of the BioGPS cutoff. (A) and (B) display the odds ratio and the P-value of a right-tailed Fisher's exact test where the contingency table counts pairs of similar (above cutoff) and different cavities containing and not containing the same ligand. Correspondingly, in (C) the G-score (harmonic mean between precision and recall) is shown. (D) Proportion of ligand volume overlapped by cavities. (E) Percentage of surface residues that are part of a cavity. (F) Distribution of all-vs-all BioGPS scores. In the pie chart, proportion of cavities for which a similar cavity is found in another structure of the same protein, in another structure of the same fold (3 ECOD levels, from specific, in dark, to more general, in light), or elsewhere. Only cavities with at least one similar cavity are counted. (G) BioGPS sensitivity, measured in cavities of different structures of the same protein. For each cavity in each structure, we looked for its partner in the second structure, and measured the BioGPS similarity of the cavity pair (only pairs of structures having almost-full protein coverage (>80%) were considered). As it can be seen in the red line, corresponding cavities in different structural instances of the same protein tend to have high BioGPS scores. Roughly, two-thirds of the cavity pairs have scores above 0.6 (dashed line). (H) Correlation between kinase inhibition profiles and cavity similarity among kinases. We downloaded a kinase-inhibitor panel from Davis et al. 2011, and exhaustively compared the ligand profile of each pair of kinases (Jaccard index of shared inhibitors). As it can be seen in red, when two kinases have similar cavities, they tend to share more ligands. (I) Top-occurring ligands in the PDB. The word-cloud displays ligands that are detected inside a cavity in at least 5 distinct proteins. These ubiquitous ligands are usually crystallographic artifacts/solvents or nature(-derived) ligands.
(TIF)

S2 Fig Background adjustments of SEA on ChEMBL. A raw score to measure the coincidence between two sets of ligands is calculated after a pairwise ligand comparison by summing up the Tanimoto coefficient (Tc) of those pairs of ligands with a Tc > 0.55. In (A) we show the background mean of the raw score at different set × set sizes, and in (B) the standard deviation (SD). In (C) we display the corresponding background Z-score distribution, fitted to an extreme-value distribution (EVD). (D) Scheme of an alternative method to SEA, involving a Naïve Bayes (NB) multi-target classification, trained on ChEMBL data, followed by a protein-protein comparison based on predicted ligand profiles (Jaccard index). (E) The enrichment of this Jaccard when we look at SEA-, fold-, sequence- and cavity-based protein pairs, compared to the background. SEA is most similar to NB, and NB shows comparable enrichments to those seen from SEA in Fig 1C in the main text (fold ~ sequence >> cavity). (F) NB-score of fold, sequence and cavity pairs, relative to SEA pairs. They are always below 1, confirming that NB and SEA are best correlated.
(TIF)

S3 Fig Therapy- and tumor-specific networks. (A) The therapeutic network of antithrombotic agents (B01), where seed nodes are highlighted in red. (B) The network of esophageal carcinoma (ESCA). In (C) and (D) we display, respectively, B01 and ESCA recall curves in a 10-fold cross-validation of the inclusion of nodes, based on the DIAMOnD algorithm. The dark line represents the recall of seed nodes, while the light line displays the proportion of seed nodes in the major component of the network.
(TIF)

S4 Fig Heat distribution analysis. (A) and (B) show the adjustments of the β parameter. When β = 1, no heat is transferred from one node to another, and at β = 0 all of the heat is released. Kidney renal cell carcinoma (KIRC) and sex hormones and modulators of the genital system (G03) networks are taken as examples to show the selection of the optimal β for each network. In (A), the network-based influence distribution on distance-one neighbors of randomly selected nodes rapidly decays at different influence inflection points, for a given β. In (B), the average inflection point at each β is displayed, and at the optimal β this inflection point is maximized. Once β is selected, to model a multi-target modulation 1,000 h.u. are distributed to the corresponding nodes and Hotnet is run. In (C) we show the distribution of heat across all nodes of the G03 and KIRC networks after a 2-node and a 3-node interference, respectively (see networks in (E)). The area under these curves is normalized by the ideal multi-target intervention, where we do a uniform assignment of heat to each of the nodes. In (D) we show that on average for all networks it is more efficient, in terms of heat distribution, to intervene with multiple targets. Finally, in (F) we demonstrate that successful targets of targeted therapies, on the corresponding tumors, do indeed distribute heat better than a random interference. To embed all networks in the same distribution, we defined a Z-score by calculating the mean and standard deviation of the heat distributed by individual random nodes. The Z-score is mostly positive, meaning that for most networks the targets of approved drugs have prominent heat-distribution properties.
(TIF)

S5 Fig Structural coverage of therapy- and tumor-specific networks. (A) Number of proteins with structural information in the different networks that we studied. For comparison, the coverage of the background proteome and known drug targets is also displayed. The structural coverage of our PPI networks is relatively high, probably due to the intrinsic bias of the Y2H PPI detection technique, which does not work well with membrane proteins. Membrane proteins are, in turn, underrepresented in the PDB. (B) Sequence coverage of the structures. Our networks contain a considerable proportion of partial structures. Data are obtained from Interactome3D.
(TIF)

## References

[1] Hopkins AL, Mason JS, Overington JP. Can we rationally design promiscuous drugs? Curr Opin Struct Biol. 2006;16(1):127–36. 10.1016/j.sbi.2006.01.013 . 16442279

[2] Hopkins AL. Network pharmacology: the next paradigm in drug discovery. Nat Chem Biol. 2008;4(11):682–90. 10.1038/nchembio.118 . 18936753

[3] Bolognesi ML. Polypharmacology in a single drug: multitarget drugs. Curr Med Chem. 2013;20(13):1639–45. . 23410164 10.2174/0929867311320130004

[4] Jalencas X, Mestres J. Identification of Similar Binding Sites to Detect Distant Polypharmacology. Mol Inform. 2013;32(11–12):976–90. 10.1002/minf.201300082 . 27481143

[5] Antolin AA, Jalencas X, Yelamos J, Mestres J. Identification of pim kinases as novel targets for PJ34 with confounding effects in PARP biology. ACS Chem Biol. 2012;7(12):1962–7. 10.1021/cb300317y . 23025350

[6] Lin H, Sassano MF, Roth BL, Shoichet BK. A pharmacological organization of G protein-coupled receptors. Nat Methods. 2013;10(2):140–6. 10.1038/nmeth.2324 . 23291723 PMC3560304

[7] Lin X, Huang XP, Chen G, Whaley R, Peng S, Wang Y, et al Life beyond kinases: structure-based discovery of sorafenib as nanomolar antagonist of 5-HT receptors. J Med Chem. 2012;55(12):5749–59. 10.1021/jm300338m . 22694093 PMC3402552

[8] Prati F, De Simone A, Bisignano P, Armirotti A, Summa M, Pizzirani D, et al Multitarget drug discovery for Alzheimer's disease: triazinones as BACE-1 and GSK-3beta inhibitors. Angew Chem Int Ed Engl. 2015;54(5):1578–82. 10.1002/anie.201410456 . 25504761

[9] Ember SW, Zhu JY, Olesen SH, Martin MP, Becker A, Berndt N, et al Acetyl-lysine binding site of bromodomain-containing protein 4 (BRD4) interacts with diverse kinase inhibitors. ACS Chem Biol. 2014;9(5):1160–71. 10.1021/cb500072z . 24568369 PMC4032195

[10] Yildirim MA, Goh KI, Cusick ME, Barabasi AL, Vidal M. Drug-target network. Nat Biotechnol. 2007;25(10):1119–26. 10.1038/nbt1338 . 17921997

[11] Lee SJ, Wang JY. Exploiting the promiscuity of imatinib. J Biol. 2009;8(3):30 10.1186/jbiol134 . 19435483 PMC2689438

[12] Duran-Frigola M, Mosca R, Aloy P. Structural systems pharmacology: the role of 3D structures in next-generation drug development. Chem Biol. 2013;20(5):674–84. 10.1016/j.chembiol.2013.03.004 . 23706634

[13] Zhou H, Gao M, Skolnick J. Comprehensive prediction of drug-protein interactions and side effects for the human proteome. Sci Rep. 2015;5:11090 10.1038/srep11090 . 26057345 PMC4603786

[14] Siragusa L, Cross S, Baroni M, Goracci L, Cruciani G. BioGPS: navigating biological space to predict polypharmacology, off-targeting, and selectivity. Proteins. 2015;83(3):517–32. 10.1002/prot.24753 . 25556939

[15] Siragusa L, Luciani R, Borsari C, Ferrari S, Costi MP, Cruciani G, et al Comparing Drug Images and Repurposing Drugs with BioGPS and FLAPdock: The Thymidylate Synthase Case. ChemMedChem. 2016;11(15):1653–66. 10.1002/cmdc.201600121 . 27404817

[16] Konc J, Janezic D. Binding site comparison for function prediction and pharmaceutical discovery. Curr Opin Struct Biol. 2014;25:34–9. 10.1016/j.sbi.2013.11.012 . 24878342

[17] Xie L, Xie L, Bourne PE. Structure-based systems biology for analyzing off-target binding. Curr Opin Struct Biol. 2011;21(2):189–99. 10.1016/j.sbi.2011.01.004 . 21292475 PMC3070778

[18] Wong MT, Choi SB, Kuan CS, Chua SL, Chang CH, Normi YM, et al Structural modeling and biochemical characterization of recombinant KPN_02809, a zinc-dependent metalloprotease from Klebsiella pneumoniae MGH 78578. Int J Mol Sci. 2012;13(1):901–17. 10.3390/ijms13010901 . 22312293 PMC3269727

[19] Rubio-Perez C, Tamborero D, Schroeder MP, Antolin AA, Deu-Pons J, Perez-Llamas C, et al In silico prescription of anticancer drugs to cohorts of 28 tumor types reveals targeting opportunities. Cancer Cell. 2015;27(3):382–96. 10.1016/j.ccell.2015.02.007 . 25759023

[20] Yizhak K, Gaude E, Le Devedec S, Waldman YY, Stein GY, van de Water B, et al Phenotype-based cell-specific metabolic modeling reveals metabolic liabilities of cancer. Elife. 2014;3 10.7554/eLife.03641 . 25415239 PMC4238051

[21] Ferrario V, Siragusa L, Ebert C, Baroni M, Foscato M, Cruciani G, et al BioGPS descriptors for rational engineering of enzyme promiscuity and structure based bioinformatic analysis. PLoS One. 2014;9(10):e109354 10.1371/journal.pone.0109354 . 25353170 PMC4212942

[22] Davis MI, Hunt JP, Herrgard S, Ciceri P, Wodicka LM, Pallares G, et al Comprehensive analysis of kinase inhibitor selectivity. Nat Biotechnol. 2011;29(11):1046–51. 10.1038/nbt.1990 . 22037378

[23] Gao M, Skolnick J. A comprehensive survey of small-molecule binding pockets in proteins. PLoS Comput Biol. 2013;9(10):e1003302 10.1371/journal.pcbi.1003302 . 24204237 PMC3812058

[24] Anastassiadis T, Deacon SW, Devarajan K, Ma H, Peterson JR. Comprehensive assay of kinase catalytic activity reveals features of kinase inhibitor selectivity. Nat Biotechnol. 2011;29(11):1039–45. 10.1038/nbt.2017 . 22037377 PMC3230241

[25] Verma N, Rai AK, Kaushik V, Brunnert D, Chahar KR, Pandey J, et al Identification of gefitinib off-targets using a structure-based systems biology approach; their validation with reverse docking and retrospective data mining. Sci Rep. 2016;6:33949 10.1038/srep33949 . 27653775 PMC5032012

[26] Capra JA, Laskowski RA, Thornton JM, Singh M, Funkhouser TA. Predicting protein ligand binding sites by combining evolutionary sequence conservation and 3D structure. PLoS Comput Biol. 2009;5(12):e1000585 10.1371/journal.pcbi.1000585 . 19997483 PMC2777313

[27] Brinkworth RI, Breinl RA, Kobe B. Structural basis and prediction of substrate specificity in protein serine/threonine kinases. Proc Natl Acad Sci U S A. 2003;100(1):74–9. 10.1073/pnas.0134224100 . 12502784 PMC140887

[28] Gregori-Puigjane E, Mestres J. Coverage and bias in chemical library design. Curr Opin Chem Biol. 2008;12(3):359–65. 10.1016/j.cbpa.2008.03.015 . 18423416

[29] DiMasi JA, Faden LB. Competitiveness in follow-on drug R&D: a race or imitation? Nat Rev Drug Discov. 2011;10(1):23–7. 10.1038/nrd3296 . 21151030

[30] Bellis LJ, Akhtar R, Al-Lazikani B, Atkinson F, Bento AP, Chambers J, et al Collation and data-mining of literature bioactivity data for drug discovery. Biochem Soc Trans. 2011;39(5):1365–70. 10.1042/BST0391365 . 21936816

[31] Keiser MJ, Irwin JJ, Shoichet BK. The chemical basis of pharmacology. Biochemistry. 2010;49(48):10267–76. 10.1021/bi101540g . 21058655 PMC2994275

[32] Lounkine E, Keiser MJ, Whitebread S, Mikhailov D, Hamon J, Jenkins JL, et al Large-scale prediction and testing of drug activity on side-effect targets. Nature. 2012;486(7403):361–7. 10.1038/nature11159 . 22722194 PMC3383642

[33] Mestres J, Gregori-Puigjane E, Valverde S, Sole RV. Data completeness—the Achilles heel of drug-target networks. Nat Biotechnol. 2008;26(9):983–4. 10.1038/nbt0908-983 . 18779805

[34] Keiser MJ, Roth BL, Armbruster BN, Ernsberger P, Irwin JJ, Shoichet BK. Relating protein pharmacology by ligand chemistry. Nat Biotechnol. 2007;25(2):197–206. 10.1038/nbt1284 . 17287757

[35] Venkatakrishnan AJ, Deupi X, Lebon G, Tate CG, Schertler GF, Babu MM. Molecular signatures of G-protein-coupled receptors. Nature. 2013;494(7436):185–94. 10.1038/nature11896 . 23407534

[36] Jia J, Zhu F, Ma X, Cao Z, Li Y, Chen YZ. Mechanisms of drug combinations: interaction and network perspectives. Nat Rev Drug Discov. 2009;8(2):111–28. 10.1038/nrd2683 . 19180105

[37] Borisy AA, Elliott PJ, Hurst NW, Lee MS, Lehar J, Price ER, et al Systematic discovery of multicomponent therapeutics. Proc Natl Acad Sci U S A. 2003;100(13):7977–82. 10.1073/pnas.1337088100 . 12799470 PMC164698

[38] Lehar J, Krueger AS, Avery W, Heilbut AM, Johansen LM, Price ER, et al Synergistic drug combinations tend to improve therapeutically relevant selectivity. Nat Biotechnol. 2009;27(7):659–66. 10.1038/nbt.1549 . 19581876 PMC2708317

[39] Rolland T, Tasan M, Charloteaux B, Pevzner SJ, Zhong Q, Sahni N, et al A proteome-scale map of the human interactome network. Cell. 2014;159(5):1212–26. 10.1016/j.cell.2014.10.050 . 25416956 PMC4266588

[40] Gu J, Zhang X, Ma Y, Li N, Luo F, Cao L, et al Quantitative modeling of dose-response and drug combination based on pathway network. J Cheminform. 2015;7:19 10.1186/s13321-015-0066-6 . 26101547 PMC4476235

[41] Liu Y, Wei Q, Yu G, Gai W, Li Y, Chen X. DCDB 2.0: a major update of the drug combination database. Database (Oxford). 2014;2014:bau124 10.1093/database/bau124 . 25539768 PMC4275564

[42] Becker E, Robisson B, Chapple CE, Guenoche A, Brun C. Multifunctional proteins revealed by overlapping clustering in protein interaction network. Bioinformatics. 2012;28(1):84–90. 10.1093/bioinformatics/btr621 . 22080466 PMC3244771

[43] Korcsmaros T, Szalay MS, Bode C, Kovacs IA, Csermely P. How to design multi-target drugs. Expert Opin Drug Discov. 2007;2(6):799–808. 10.1517/17460441.2.6.799 . 23488998

[44] Csermely P, Agoston V, Pongor S. The efficiency of multi-target drugs: the network approach might help drug design. Trends Pharmacol Sci. 2005;26(4):178–82. 10.1016/j.tips.2005.02.007 . 15808341

[45] Yang K, Bai H, Ouyang Q, Lai L, Tang C. Finding multiple target optimal intervention in disease-related molecular network. Mol Syst Biol. 2008;4:228 10.1038/msb.2008.60 . 18985027 PMC2673713

[46] Ghiassian SD, Menche J, Barabasi AL. A DIseAse MOdule Detection (DIAMOnD) algorithm derived from a systematic analysis of connectivity patterns of disease proteins in the human interactome. PLoS Comput Biol. 2015;11(4):e1004120 10.1371/journal.pcbi.1004120 . 25853560 PMC4390154

[47] Azuaje F, Devaux Y, Wagner DR. Identification of potential targets in biological signalling systems through network perturbation analysis. Biosystems. 2010;100(1):55–64. 10.1016/j.biosystems.2010.01.002 . 20079399

[48] Yadav G, Babu S. NEXCADE: perturbation analysis for complex networks. PLoS One. 2012;7(8):e41827 10.1371/journal.pone.0041827 . 22870252 PMC3411682

[49] Leiserson MD, Vandin F, Wu HT, Dobson JR, Eldridge JV, Thomas JL, et al Pan-cancer network analysis identifies combinations of rare somatic mutations across pathways and protein complexes. Nat Genet. 2015;47(2):106–14. 10.1038/ng.3168 . 25501392 PMC4444046

[50] Barabasi AL, Gulbahce N, Loscalzo J. Network medicine: a network-based approach to human disease. Nat Rev Genet. 2011;12(1):56–68. 10.1038/nrg2918 . 21164525 PMC3140052

[51] Bordbar A, Monk JM, King ZA, Palsson BO. Constraint-based models predict metabolic and associated cellular functions. Nat Rev Genet. 2014;15(2):107–20. 10.1038/nrg3643 . 24430943

[52] Orth JD, Thiele I, Palsson BO. What is flux balance analysis? Nat Biotechnol. 2010;28(3):245–8. 10.1038/nbt.1614 . 20212490 PMC3108565

[53] Cairns RA, Harris IS, Mak TW. Regulation of cancer cell metabolism. Nat Rev Cancer. 2011;11(2):85–95. 10.1038/nrc2981 . 21258394

[54] Schomburg I, Chang A, Ebeling C, Gremse M, Heldt C, Huhn G, et al BRENDA, the enzyme database: updates and major new developments. Nucleic Acids Res. 2004;32(Database issue):D431–3. 10.1093/nar/gkh081 . 14681450 PMC308815

[55] Haupt VJ, Schroeder M. Old friends in new guise: repositioning of known drugs with structural bioinformatics. Brief Bioinform. 2011;12(4):312–26. 10.1093/bib/bbr011 . 21441562

[56] Mosca R, Pons T, Ceol A, Valencia A, Aloy P. Towards a detailed atlas of protein-protein interactions. Curr Opin Struct Biol. 2013;23(6):929–40. 10.1016/j.sbi.2013.07.005 . 23896349

[57] Sethi G, Chopra G, Samudrala R. Multiscale modelling of relationships between protein classes and drug behavior across all diseases using the CANDO platform. Mini Rev Med Chem. 2015;15(8):705–17. . 25694071 10.2174/1389557515666150219145148 PMC5903852

[58] Mosca R, Ceol A, Aloy P. Interactome3D: adding structural details to protein networks. Nat Methods. 2013;10(1):47–53. 10.1038/nmeth.2289 . 23399932

[59] Lajiness MS, Vieth M, Erickson J. Molecular properties that influence oral drug-like behavior. Curr Opin Drug Discov Devel. 2004;7(4):470–7. . 15338956

[60] Waring MJ, Arrowsmith J, Leach AR, Leeson PD, Mandrell S, Owen RM, et al An analysis of the attrition of drug candidates from four major pharmaceutical companies. Nat Rev Drug Discov. 2015;14(7):475–86. 10.1038/nrd4609 . 26091267

[61] Besnard J, Ruda GF, Setola V, Abecassis K, Rodriguiz RM, Huang XP, et al Automated design of ligands to polypharmacological profiles. Nature. 2012;492(7428):215–20. 10.1038/nature11691 . 23235874 PMC3653568

[62] Minie M, Chopra G, Sethi G, Horst J, White G, Roy A, et al CANDO and the infinite drug discovery frontier. Drug Discov Today. 2014;19(9):1353–63. 10.1016/j.drudis.2014.06.018 . 24980786 PMC4167471

[63] Chopra G, Samudrala R. Exploring Polypharmacology in Drug Discovery and Repurposing Using the CANDO Platform. Curr Pharm Des. 2016;22(21):3109–23. . 27013226 10.2174/1381612822666160325121943 PMC6376875

[64] Goodford PJ. A computational procedure for determining energetically favorable binding sites on biologically important macromolecules. J Med Chem. 1985;28(7):849–57. . 3892003 10.1021/jm00145a002

[65] Johnson LS, Eddy SR, Portugaly E. Hidden Markov model speed heuristic and iterative HMM search procedure. BMC Bioinformatics. 2010;11:431 10.1186/1471-2105-11-431 . 20718988 PMC2931519

[66] Cheng H, Schaeffer RD, Liao Y, Kinch LN, Pei J, Shi S, et al ECOD: an evolutionary classification of protein domains. PLoS Comput Biol. 2014;10(12):e1003926 10.1371/journal.pcbi.1003926 . 25474468 PMC4256011

[67] Knox C, Law V, Jewison T, Liu P, Ly S, Frolkis A, et al DrugBank 3.0: a comprehensive resource for 'omics' research on drugs. Nucleic Acids Res. 2011;39(Database issue):D1035–41. 10.1093/nar/gkq1126 . 21059682 PMC3013709

[68] Menche J, Sharma A, Kitsak M, Ghiassian SD, Vidal M, Loscalzo J, et al Disease networks. Uncovering disease-disease relationships through the incomplete interactome. Science. 2015;347(6224):1257601 . 25700523 10.1126/science.1257601 PMC4435741

[69] Burgard AP, Pharkya P, Maranas CD. Optknock: a bilevel programming framework for identifying gene knockout strategies for microbial strain optimization. Biotechnol Bioeng. 2003;84(6):647–57. 10.1002/bit.10803 . 14595777

[70] Gene Ontology C. Gene Ontology Consortium: going forward. Nucleic Acids Res. 2015;43(Database issue):D1049–56. 10.1093/nar/gku1179 . 25428369 PMC4383973

