# Supplementary figures and images for: Detecting similar binding pockets to enable systems polypharmacology
Source: PLoS Comput Biol. 2017 Jun 29;13(6):e1005522. doi: 10.1371/journal.pcbi.1005522 (PMC5490940; doi:10.1371/journal.pcbi.1005522)

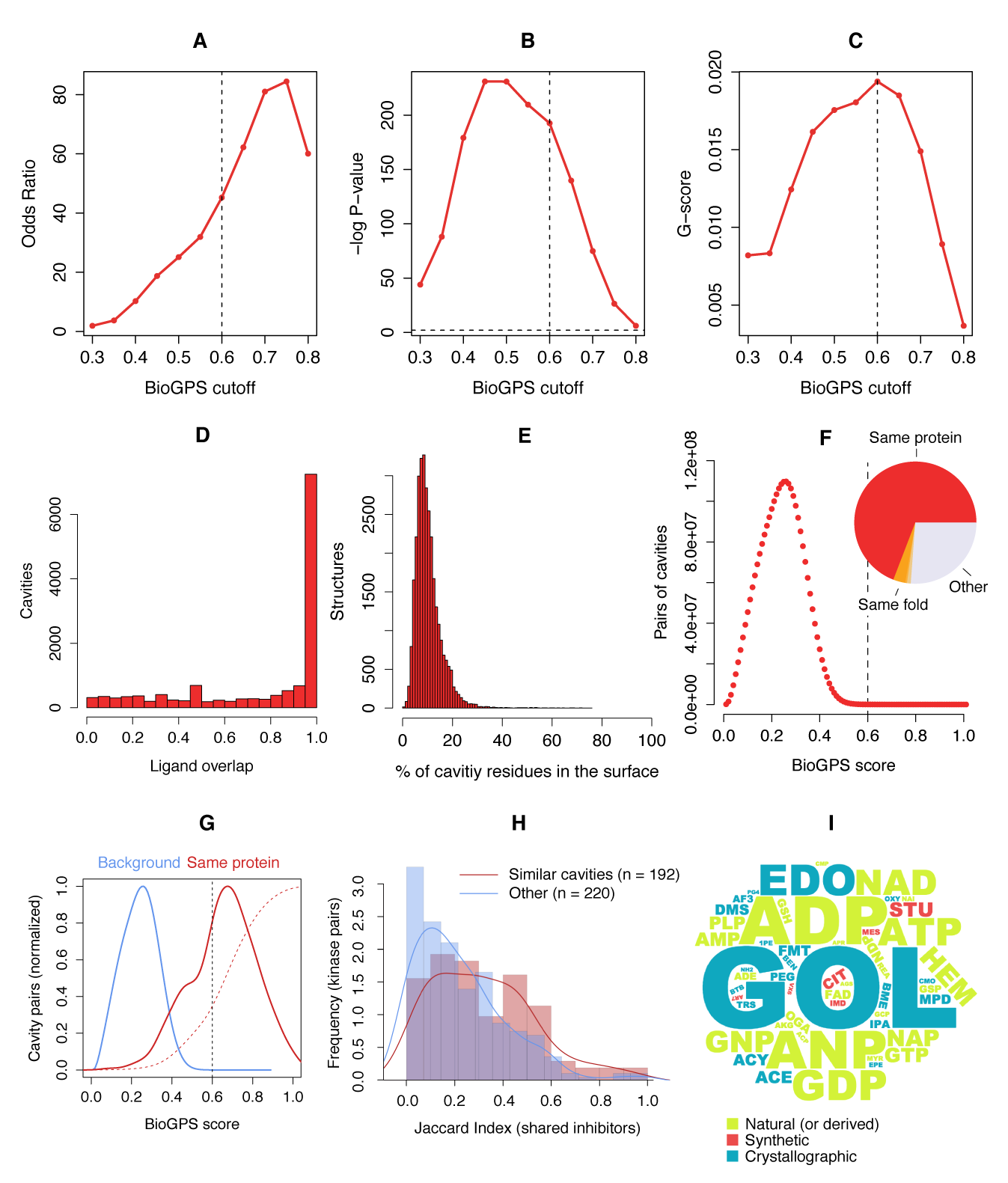

Supplement: S1 Fig — (A) and (B) display the odds ratio and the P-value of a right-tailed Fisher's exact test where the contingency table counts pairs of similar (above cutoff) and different cavities containing and not containing the same ligand. Correspondingly, in (C) the G-score (harmonic mean between precision and recall) is shown. (D) Proportion of ligand volume overlapped by cavities. (E) Percentage of surface residues that are part of a cavity. (F) Distribution of all-vs-all BioGPS scores. In the pie chart, proportion of cavities for which a similar cavity is found in another structure of the same protein, in another structure of the same fold (3 ECOD levels, from specific, in dark, to more general, in light), or elsewhere. Only cavities with at least one similar cavity are counted. (G) BioGPS sensitivity, measured in cavities of different structures of the same protein. For each cavity in each structure, we looked for its partner in the second structure, and measured the BioGPS similarity of the cavity pair (only pairs of structures having almost-full protein coverage (>80%) were considered). As it can be seen in the red line, corresponding cavities in different structural instances of the same protein tend to have high BioGPS scores. Roughly, two-thirds of the cavity pairs have scores above 0.6 (dashed line). (H) Correlation between kinase inhibition profiles and cavity similarity among kinases. We downloaded a kinase-inhibitor panel from Davis et al. 2011, and exhaustively compared the ligand profile of each pair of kinases (Jaccard index of shared inhibitors). As it can be seen in red, when two kinases have similar cavities, they tend to share more ligands. (I) Top-occurring ligands in the PDB. The word-cloud displays ligands that are detected inside a cavity in at least 5 distinct proteins. These ubiquitous ligands are usually crystallographic artifacts/solvents or nature(-derived) ligands. (TIF) [file pcbi.1005522.s002.tif]

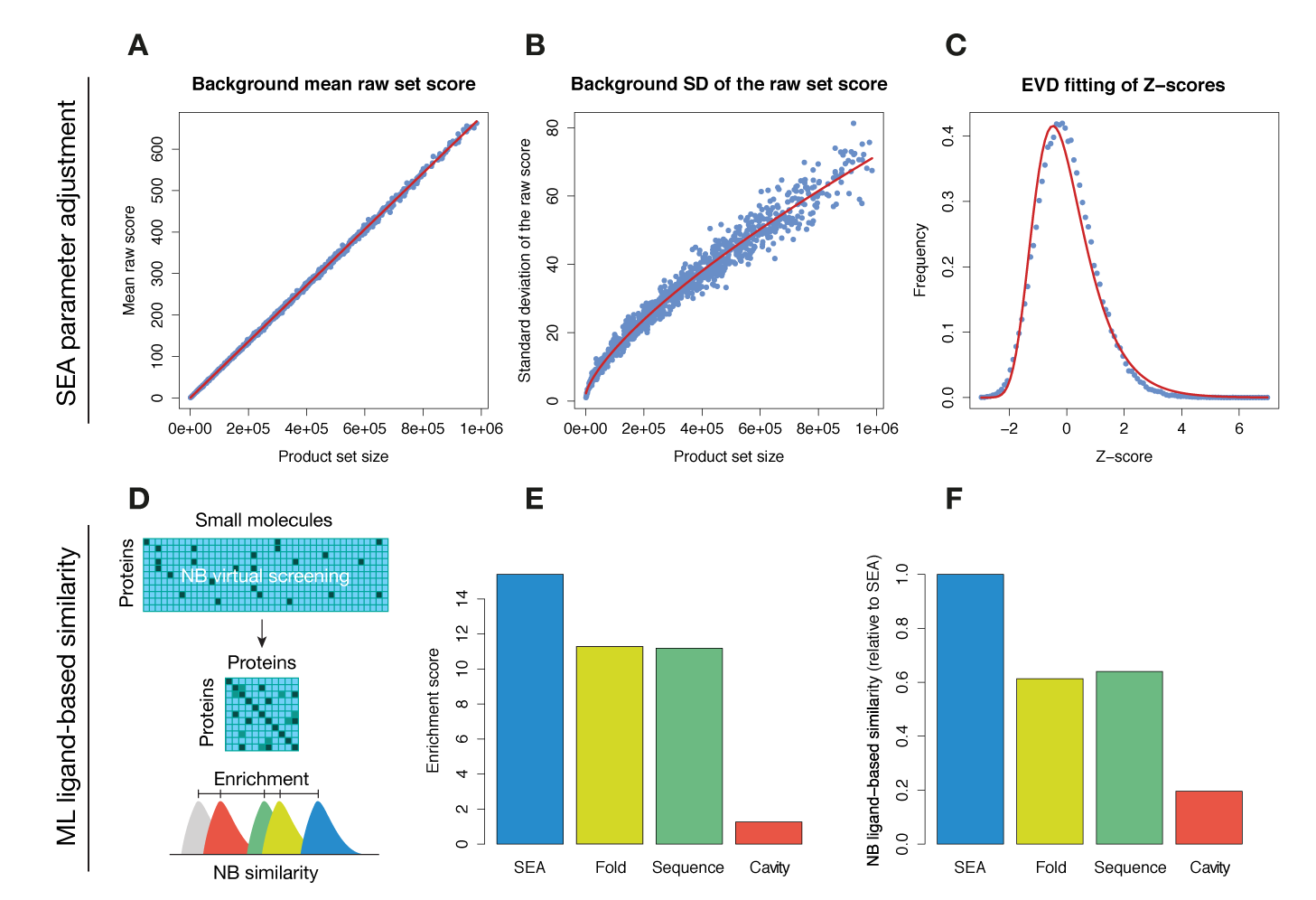

Supplement: S2 Fig — A raw score to measure the coincidence between two sets of ligands is calculated after a pairwise ligand comparison by summing up the Tanimoto coefficient (Tc) of those pairs of ligands with a Tc > 0.55. In (A) we show the background mean of the raw score at different set × set sizes, and in (B) the standard deviation (SD). In (C) we display the corresponding background Z-score distribution, fitted to an extreme-value distribution (EVD). (D) Scheme of an alternative method to SEA, involving a Naïve Bayes (NB) multi-target classification, trained on ChEMBL data, followed by a protein-protein comparison based on predicted ligand profiles (Jaccard index). (E) The enrichment of this Jaccard when we look at SEA-, fold-, sequence- and cavity-based protein pairs, compared to the background. SEA is most similar to NB, and NB shows comparable enrichments to those seen from SEA in Fig 1C in the main text (fold ~ sequence >> cavity). (F) NB-score of fold, sequence and cavity pairs, relative to SEA pairs. They are always below 1, confirming that NB and SEA are best correlated. (TIF) [file pcbi.1005522.s003.tif]

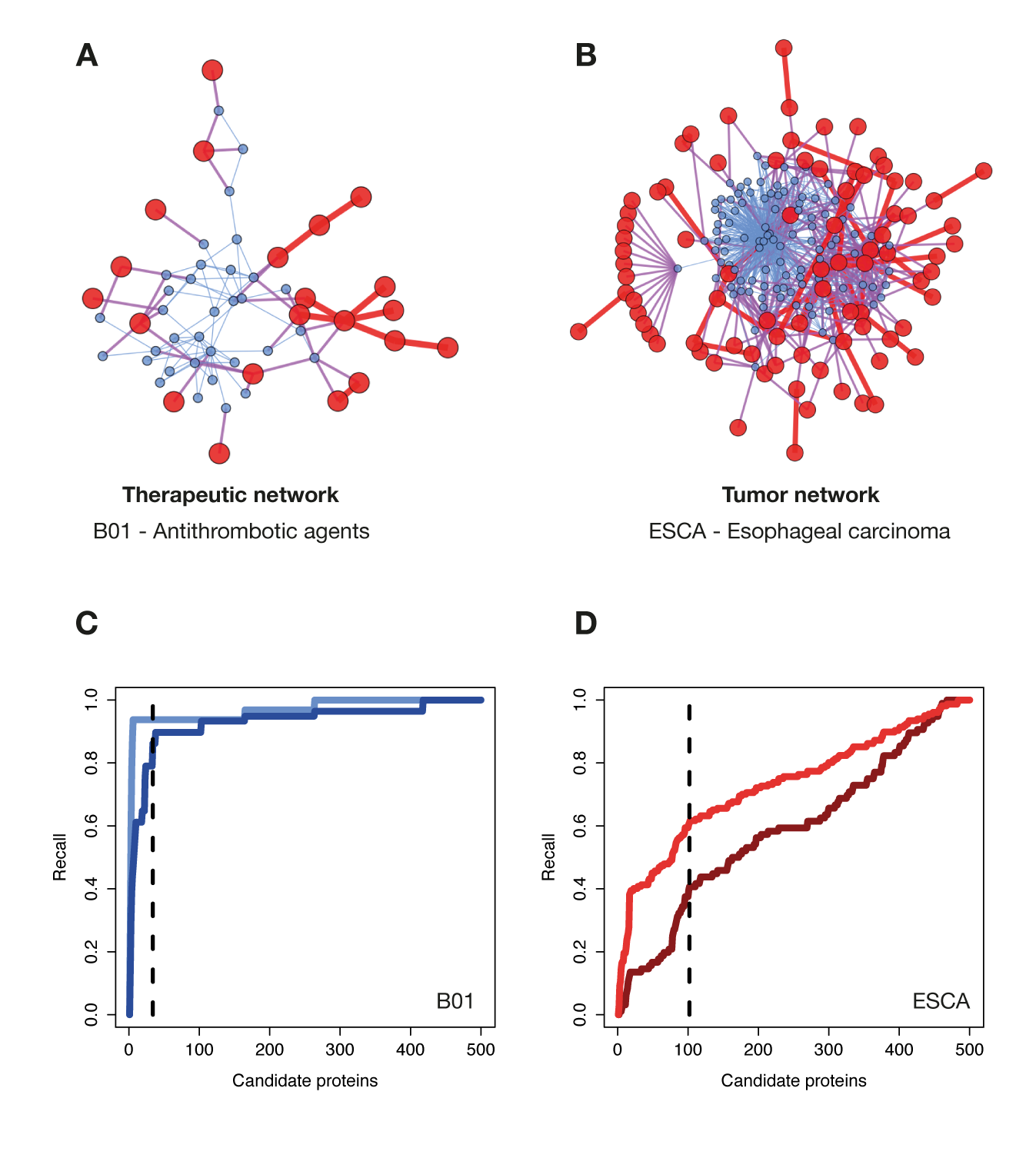

Supplement: S3 Fig — (A) The therapeutic network of antithrombotic agents (B01), where seed nodes are highlighted in red. (B) The network of esophageal carcinoma (ESCA). In (C) and (D) we display, respectively, B01 and ESCA recall curves in a 10-fold cross-validation of the inclusion of nodes, based on the DIAMOnD algorithm. The dark line represents the recall of seed nodes, while the light line displays the proportion of seed nodes in the major component of the network. (TIF) [file pcbi.1005522.s004.tif]

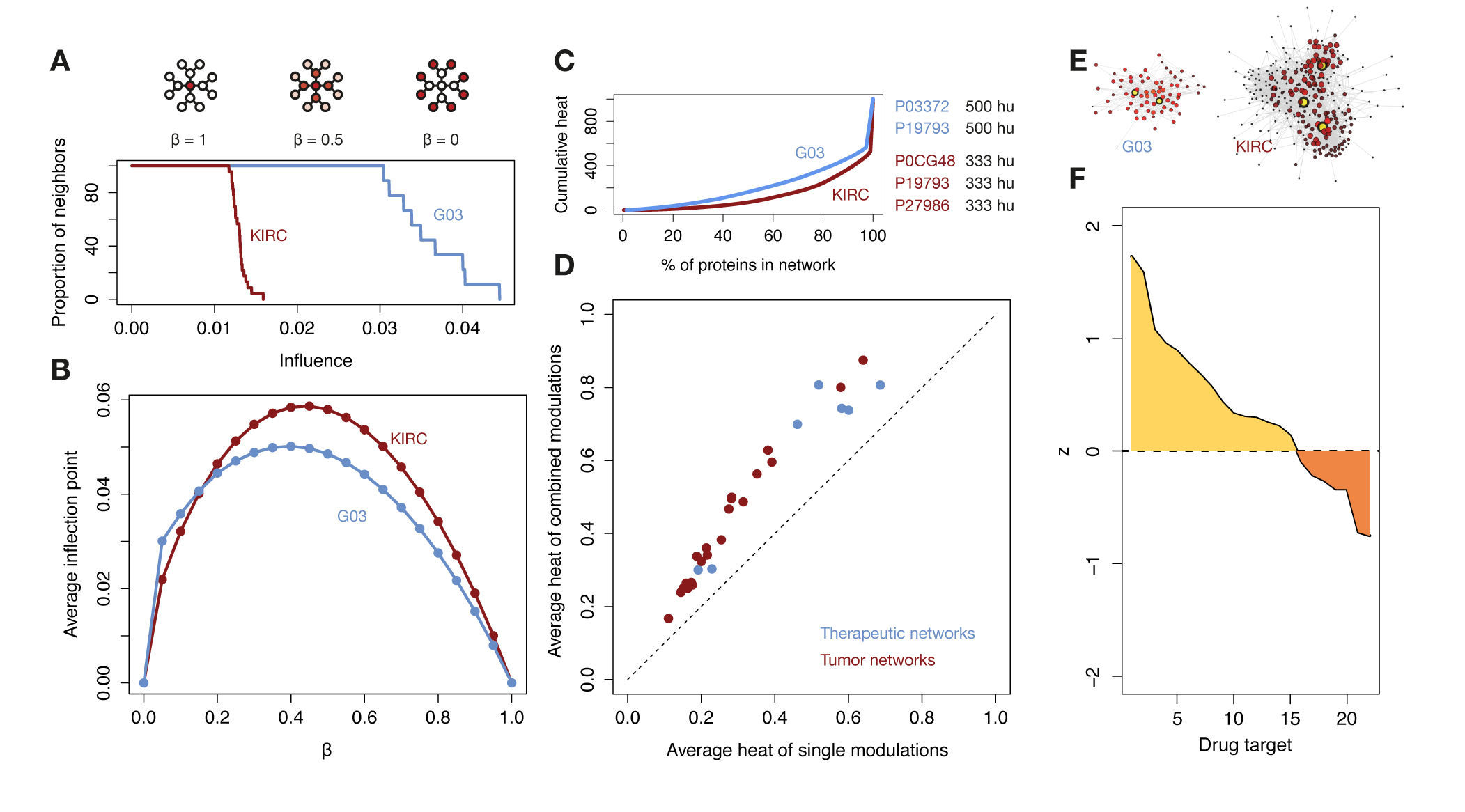

Supplement: S4 Fig — (A) and (B) show the adjustments of the β parameter. When β = 1, no heat is transferred from one node to another, and at β = 0 all of the heat is released. Kidney renal cell carcinoma (KIRC) and sex hormones and modulators of the genital system (G03) networks are taken as examples to show the selection of the optimal β for each network. In (A), the network-based influence distribution on distance-one neighbors of randomly selected nodes rapidly decays at different influence inflection points, for a given β. In (B), the average inflection point at each β is displayed, and at the optimal β this inflection point is maximized. Once β is selected, to model a multi-target modulation 1,000 h.u. are distributed to the corresponding nodes and Hotnet is run. In (C) we show the distribution of heat across all nodes of the G03 and KIRC networks after a 2-node and a 3-node interference, respectively (see networks in (E)). The area under these curves is normalized by the ideal multi-target intervention, where we do a uniform assignment of heat to each of the nodes. In (D) we show that on average for all networks it is more efficient, in terms of heat distribution, to intervene with multiple targets. Finally, in (F) we demonstrate that successful targets of targeted therapies, on the corresponding tumors, do indeed distribute heat better than a random interference. To embed all networks in the same distribution, we defined a Z-score by calculating the mean and standard deviation of the heat distributed by individual random nodes. The Z-score is mostly positive, meaning that for most networks the targets of approved drugs have prominent heat-distribution properties. (TIF) [file pcbi.1005522.s005.tif]

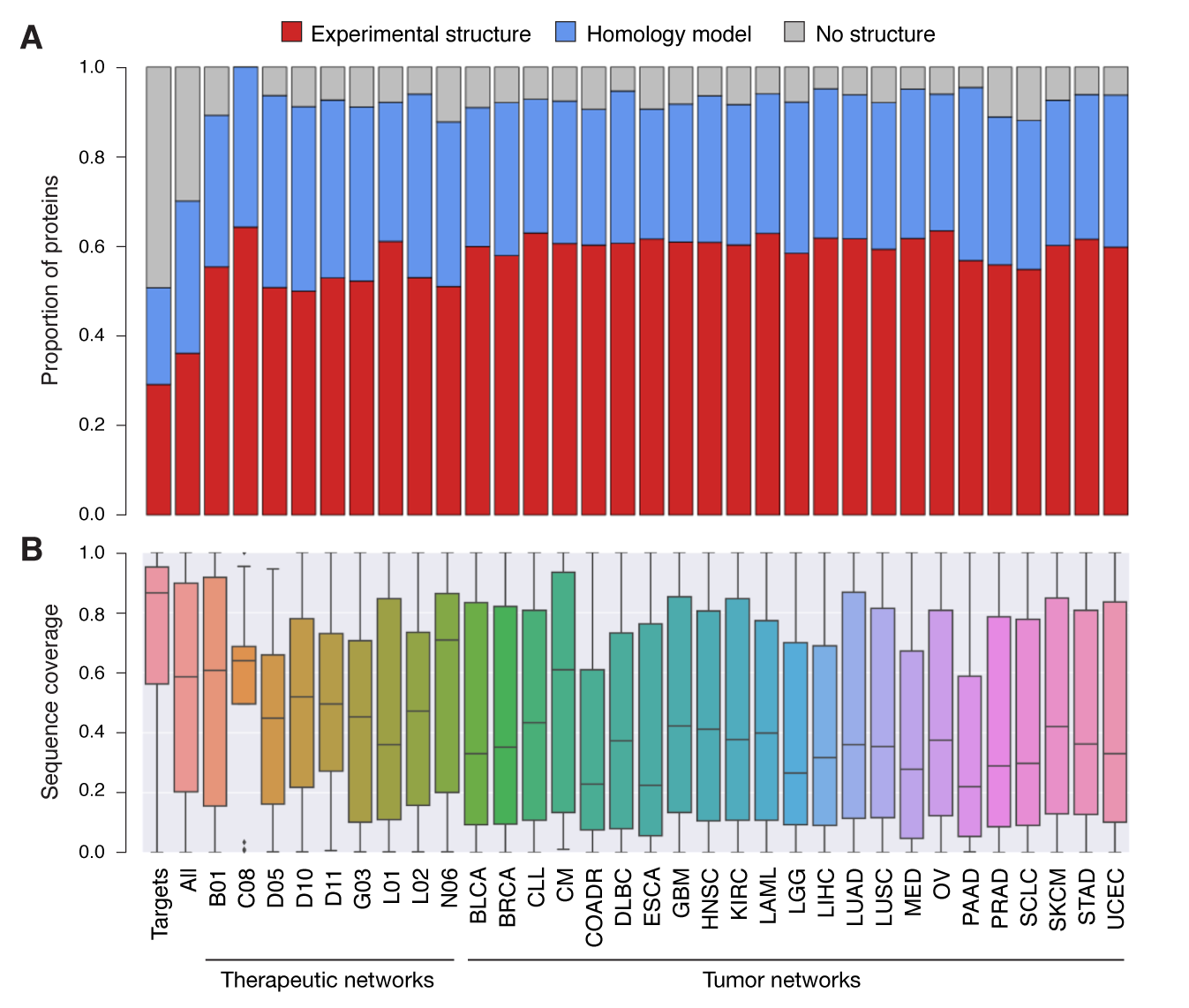

Supplement: S5 Fig — (A) Number of proteins with structural information in the different networks that we studied. For comparison, the coverage of the background proteome and known drug targets is also displayed. The structural coverage of our PPI networks is relatively high, probably due to the intrinsic bias of the Y2H PPI detection technique, which does not work well with membrane proteins. Membrane proteins are, in turn, underrepresented in the PDB. (B) Sequence coverage of the structures. Our networks contain a considerable proportion of partial structures. Data are obtained from Interactome3D. (TIF) [file pcbi.1005522.s006.tif]
